# Enhanced Fire Safety of Rigid Polyurethane Foam via Synergistic Effect of Phosphorus/Nitrogen Compounds and Expandable Graphite

**DOI:** 10.3390/molecules25204741

**Published:** 2020-10-15

**Authors:** Chuan Liu, Ping Zhang, Yongqian Shi, Xiaohui Rao, Suncheng Cai, Libi Fu, Yuezhan Feng, Liancong Wang, Xueqin Zheng, Wei Yang

**Affiliations:** 1 College of Environment and Resources, Fuzhou University, 2 Xueyuan Road, Fuzhou 350116, China; liuchuanll@163.com (C.L.); Rxh123456789m88@163.com (X.R.); dandy_cai@163.com (S.C.); 2 State key Laboratory of Enviromental Friendly Energy Materials & Department of Materials, Southwest University of Science and Technology, Mianyang 621010, China; pingzhang@swust.edu.cn; 3 College of Civil Engineering, Fuzhou University, 2 Xueyuan Road, Fuzhou 350116, China; fulibi@fzu.edu.cn; 4 Key Laboratory of Materials Processing and Mold Ministry of Education, National Engineering Research Center for Advanced Polymer Processing Technology, Zhengzhou University, Zhengzhou 450002, China; yzfeng@zzu.edu.cn; 5 State Key Laboratory of Coal Mine Safety Technology, CCTEG Shenyang Research Institute, Fushun 113122, China; 6 College of Safety and Environment, Fujian Chuanzheng Communications College, 80 Shoushan Road, Fuzhou 350007, China; zhengxueqin815@foxmail.com; 7 School of Energy, Materials and Chemical Engineering, Hefei University, Hefei 230601, China

**Keywords:** flame retardancy, rigid polyurethane foam, phosphorus-nitrogen compounds, heat release, synergistic effect

## Abstract

In order to explore highly efficient flame-retardant rigid polyurethane foam (RPUF), phosphorus/nitrogen compounds and expandable graphite (EG) were successfully incorporated into RPUF by a free one-spot method. The combustion results showed that the fire safety of the RPUF samples was remarkably improved by the addition of phosphoric/nitrogen compounds and EG. With the incorporation of 22.4 wt.% phosphorus/nitrogen compounds and 3.2 wt.% EG, the RPUF composites achieved UL-94 V-0 rating. Besides, the total heat release and total smoke release of RPUF composites were reduced by 29.6% and 32.4% respectively, compared to those of the pure RPUF sample. PO• and PO_2_• together with nonflammable gaseous products were evolved from phosphoric/nitrogen compounds in the gas phase, which quenched the flammable free radicals in the matrix and diluted the concentration of combustible gaseous products generated from PRUF during combustion. The compact char residues which acted as excellent physical barriers were formed by catalysis of EG and phosphoric/nitrogen compounds in the condense phase. The fire hazard of RPUF was significantly reduced by the synergistic effect of phosphorus-nitrogen compounds and EG. This work provides a promising strategy to enhance the fire safety of RPUF.

## 1. Introduction

Polyurethanes (PUs) have been widely utilized in various fields including paints, construction, adhesives, refrigeration, aeronautics, medical devices, sealants and oil pipeline [1,2,3,4]. Rigid polyurethane foam (RPUF) is one of the most extensively applied PU materials due to its excellent mechanical and adiabatic properties, such as high compressive strength, light weight, construction convenience, low water absorption, low thermal conductivity and so on [5,6,7]. However, during combustion, RPUF will generate excessive heat, poisonous smoke and toxic gases, such as nitrogen oxide (NOx), carbon monoxide (CO), hydrogen cyanide (HCN) and so on. This will cause numerous deaths and serious pollution for the environment. Therefore, it is significant to improve the fire safety of RPUF for broadening the application of RPUF in multifarious fields [8,9,10].

To date, halogen-free flame retardants including ammonium polyphosphate (APP) [11,12,13], triphenyl phosphate (TPP) [14], modified montmorillonite (MMT) [15], melamine and its salts [16,17,18], aluminum hydroxide (ATH) [19,20,21] and aluminum hypophosphite (AHP) [22,23,24], etc., have been widely used in RPUF due to its low-cost, environmentally friendly and highly flame-retardant effective properties. Phosphorus-nitrogen (P/N) flame retardant is one of the excellent halogen-free flame retardants due to the prominent flame-retardant efficiency. After the addition of P/N flame retardants, POx and NOx free radicals were released in the gas phase during burning, which dilute the concentration of combustible gases and discontinue the free radical chain reaction of combustion [25,26]. Based on previous reports, 2-carboxyethyl phenylphosphinic acid glycol ester (CEPPG), melamine phosphate (MP) and melamine phosphite (MPi) were efficient P/N flame retardants, which were endowed with the high content of nitrogen and phosphorous elements [27,28,29]. However, those P/N flame retardants produce slight and flurry char residues during combustion, which leads to the poor smoke suppression. Thus, it is significant to combine those P/N flame retardants with highly efficient carbon source to enhance the property of char residues.

Expandable graphite (EG), acting as an excellent halogen-free flame retardant with a highly carbon source, generates a worm-like char layer during its combustion. This compact and firm char layer has a distinguished obstructing effect on smoke and heat. As a prominent flame retardant, EG was universally applied in various polymeric materials, especially in RPUF [30]. Wang et al. introduced 6.67 wt.% EG and 13.33 wt.% MP into polystyrene and found that the peak of heat release (PHRR) was reduced by 40.4% [31]. Pang et al. have incorporated three different sizes of tripolyphosphate-modified expandable graphite (EGp) into RPUF. As the size of EGp increases, RPUFs showed an increased LOI (limiting oxygen index) and a decreased heat release [32]. Chen et al. introduced 15 wt.% EG into RPUF and found that the PHRR and total heat release (THR) were decreased by 36.2% and 22.0%, respectively [33]. Besides, EG is used to combine with P/N flame retardants for highly efficient flame retardancy of the RPUF. Xi et al. prepared a flame-retardant RPUF with [bis(2-hydroxyethyl)amino]-methyl-phos-phonic acid dimethyl ester (BH)/EG by the box-foaming approach and found that THR and total smoke release (TSR) of RPUF containing 12 wt.% BH and 8 wt.% EG were respectively decreased by 31.0% and 41.3% [34]. Wang et al. fabricated the flame-retardant RPUF by addition of pentaerythritol phosphate (PEPA) and EG [35]. The PHRR of RPUF sample with the incorporation of 20.0 wt.% PEPA/EG was decreased by 65.1%. A reactive phenylphosphoryl glycol ether oligomer (PPGE) and EG was synthesized by Wu et al., and the THR and TSR of RPUF were significantly decreased with the addition of PPGE and EG [36]. Feng et al. found that the TSR of the RPUF sample was decreased by 69.9% with the addition of 14 wt.% dimethyl methylphosphonate/graphite [37]. However, scarcely any previous work focuses on the synergistic flame-retardant effect among EG, CEPPG and MPi (or MP). It is anticipated that those P/N flame retardants can play a significant role in the gas phase and EG can act as firm char residues in the condensed phase. Therefore, a highly efficient flame retardancy should be obtained in the synergistic effect between P/N flame retardants and EG.

In this work, an intumescent flame-retardant system consisting of EG and different chemical valence phosphorus-nitrogen flame retardants (CEPPG and MP/MPi) has been successfully incorporated into RPUF. Thermal stability and flame-retardant performances were investigated in detail. In addition, the flame-retardant mechanism was discussed. This work provides a potential method to improve the fire safety of RPUF by combining P/N flame retardants with EG.

## 2. Results and Discussion

### 2.1. Thermal Degradation Behavior Analysis

**Figure 1:**
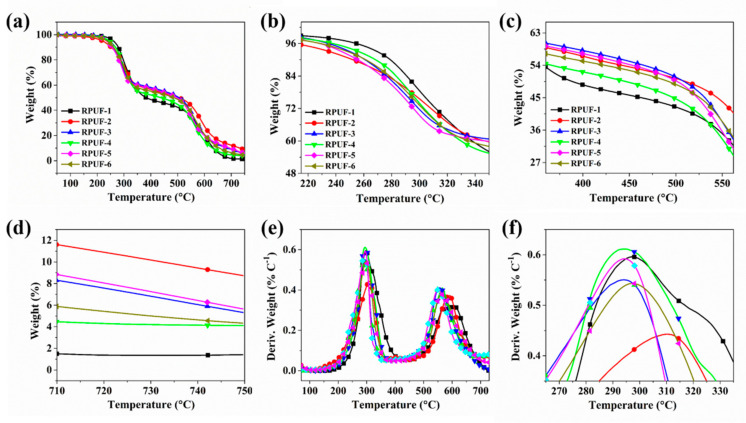
(**a**) Thermogravimetry (TG) and (**e**) derivative thermogravimetry (DTG) curves of RPUF and its composites under air condition, (**b**–**d**) are magnifications of (**a**) and (**f**) is magnifications of (**e**).

The thermal stability of polymeric materials was investigated by the TG technique [38,39]. The TG and differential thermogravimetry (DTG) curves of RPUF and its composites are portrayed in Figure 1, and the related thermal data are recorded in Table 1. As shown in Figure 1a, e, the thermal decomposition process of all the RPUF samples contains two main stages, which is in good consistency with the previous report [40]. The first decomposition stage is attributed the degradation of PU chains to form the monomeric precursor such as polyhydric alcohols, isocyanate, amines, etc., while the second degradation stage is caused by the decomposition of the substituted urea, which results from the reaction of carbodiimide with water vapor or alcohol [41,42]. As shown in Table 1, the T_−5_ values of all the RPUF composites are lower than that of pure RPUF. This is caused by the early degradation of P/N compounds. After the addition of flame retardants, the T-max of RPUF composites is also slightly decreased. It is clearly observed that the T-max of RPUF-2 is the highest among all the RPUF composites, probably due to the excellent thermal stability property of CEPPG. Although T_−5_ and T_-max_ are decreased, the char residues of all PRUF composites show an increasing tendency. For instance, the char yield of RPUF-2 is markedly increased by 520% compared to that of pure RPUF, which is attributed to the outstanding catalytic charring of CEPPG. The RPUF sample containing MP alone shows a higher char yield than RPUF filled with the equal amount of only MPi, indicating that the charring effect of MP is better than that of MPi. This phenomenon is caused by the difference of P valence between MP and MPi. Besides, it is found that with increasing of flame-retardant additives, the char residues of RPUF composites are also increased. This phenomenon demonstrated the synergistic effect between EG and P/N compounds in improved char quality. The above results indicate that the thermal stability of RPUF is improved by the synergistic effect between P/N compounds and EG.

### 2.2. Combustion Behavior Evaluation of RPUF and Its Composites

**Figure 2:**
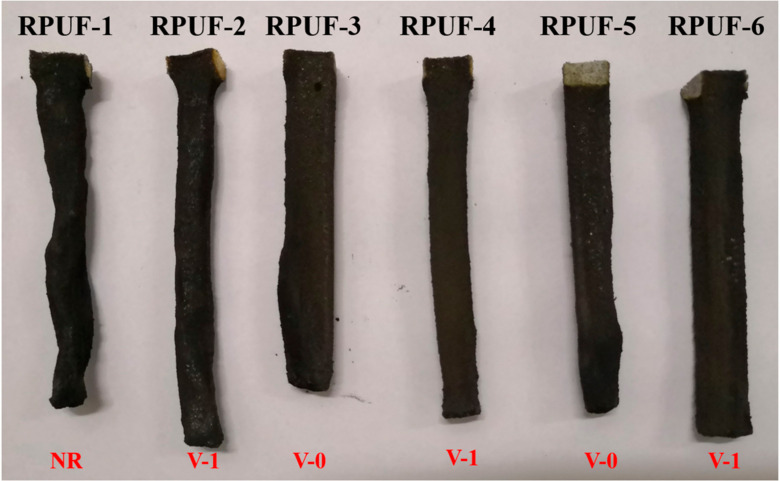
Digital photographs of char residues of RPUF and its composites after UL-94 testing.

The UL-94 vertical burning test is a universal measurement to assess the combustion property of polymeric materials [43,44,45]. The results of the UL-94 vertical burning test for RPUF and its composites are summarized in Figure 2 and Table 2. As shown in Table 2, the pristine RPUF cannot pass the UL-94 V-0 rating, resulting from its high flammability. It is observed that only a small part of material is left after the combustion of RPUF-1. After the addition of P/N compounds and EG, the RPUF composites obtain an obviously improved UL-94 rating and the self-extinguish phenomenon occurs. Besides, the char residues show a firm and intact morphology. This result is attributed to the additives promoting the formation of compact char residues which can act as the barrier to prevent the transfer of heat and mass. It is obviously observed that the RPUF-2 shows the longest t_1_ among all the RPUF composites. However, its t_1_ is decreased upon the incorporation of EG, which further demonstrates the synergistic effect between P/N compounds and EG. It is noted that both the RPUF-3 and RPUF-5 reach a UL-94 V-0 rating, whereas the RPUF-4 and RPUF-6 only pass the UL-94 V-1 testing, indicating the superior synergistic effect between the EG and MP over that between EG and MPi.

**Figure 3:**
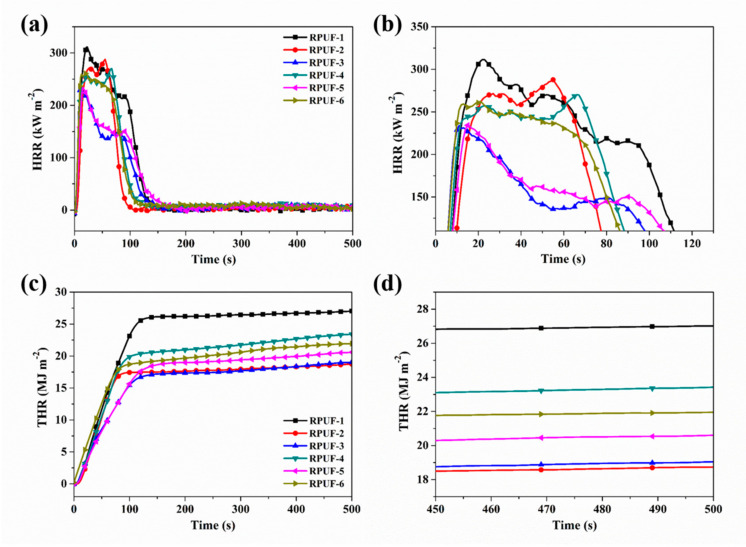
(**a**,**b**) Heat release rate (HRR) and (**c**,**d**) total heat release (THR) curves of RPUF and its composites: (**b**), (**d**) are magnifications of (**a**) and (**c**), respectively.

**Figure 4:**
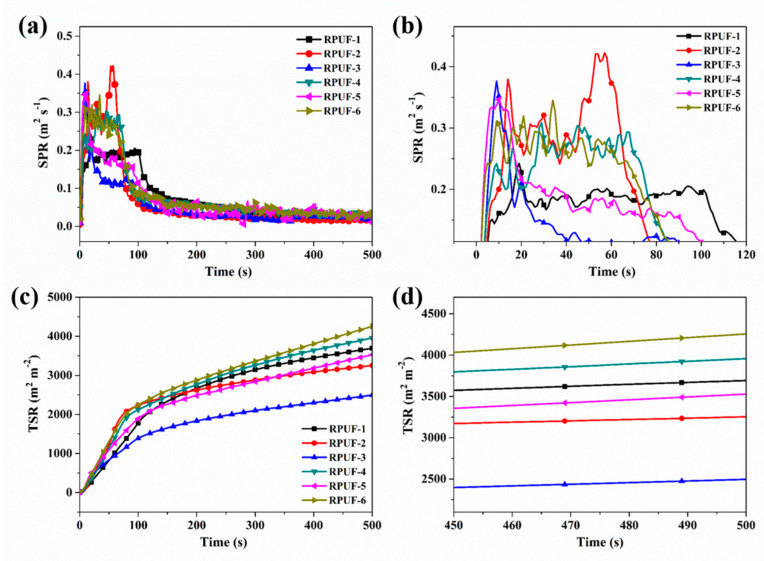
(**a**) SPR and (**c**) TSR curves of RPUF and its composites: (**b**), (**d**) are magnifications of (**a**) and (**c**), respectively.

Cone calorimeter is an effective technique to evaluate the combustion properties of the polymeric materials [46,47,48]. The results of cone calorimeter testing for RPUF and its composites are portrayed in Figure 3 and Figure 4, and corresponding cone data are listed in Table 3. It is found that all the RPUF samples show rapid combustion according to time to ignition (TTI) values. This phenomenon is attributed to the porous structures of foam materials. After the addition of P/N compounds and EG, the TTI of RPUF composites are lower than that of pure RPUF. The result is caused by the decomposition process of the P-O-C bond in the polyurethane molecular chain was promoted, which is agreement with the pervious report [49]. As shown in Figure 3a,b, two peaks are visible in the HRR cures of RPUF and its composites. The first peak is ascribed to the existence of the protective char which prevents the heat and mass transfer, whereas the second peak appears when the protective char has failed [50]. It is evident that the first PHRR of all the RPUF composites appears in advance and the second PHRR is postponed compared to those of pure RPUF. This phenomenon indicates that the solid char is formed by the introduction of flame-retardant additives. With the addition of CEPPG, the PHRR of RPUF-2 is decreased by 8.0%. The PHRR of RPUF composites further decreases after the incorporation of P/N compounds and EG. For instance, the PHRR of RPUF-3 is 233 kW m^−2^, decreased by 25.3% compared to that of pure RPUF, implying the lowest value among those of all the RPUF samples. Besides, the PHRR of RPUF-3 is lower than that of RPUF-4, indicating that the synergistic effect between MP and EG is better than that between MPi and EG. These results are in good agreement with the TGA testing. The THR of RPUF and its composites also shows the same tendency as PHRR. It is noteworthy that the THR of RPUF-3 is 19.0 MJ m^−2^, which is reduced by 29.6% compared to that of pure RPUF. However, the THR of RPUF-5 is decreased by 23.7%, due to the reason that the RPUF-5 contains excess EG and P/N compounds. These above results further indicate the synergistic flame retardancy between EG and P/N compounds.

Generally, smoke has a significant impact on the evacuation in the fire accident. Therefore, it is important to investigate the smoke release during the combustion of the polymeric materials. The SPR data of RPUF and its composites are presented in Figure 4 and Table 3. Obviously, the RPUF composites show slightly increased PSPR values compared to pure RPUF. This phenomenon is contributed to the explanation that the P-containing compounds thermally decompose to more smoke during the burning process of RPUF, which is well accordant with the previous work [51]. It is observed that the TSR value of RPUF-2 is 3255 m^2^ m^−2^, which is reduced by 12.0% compared to that of pure RPUF. After the addition of CEPPG, MPi and EG, the TSR values of RPUF composites, i.e., RPUF-4 and RPUF-6, exhibit an increase tendency. However, the TSR of RPUF composites is decreased after the incorporation of CEPPG, MP and EG. For example, the RPUF-3 shows a 32.7% reduction in TSR compared to pure RPUF, indicating the highest fire safety among all the RPUF composites. This result further indicates the superior synergistic effect of EG and MP in inhibiting the smoke release. The above results demonstrate that the flame retardancy of RPUF is improved by the addition of suitable EG and P/N compounds.

### 2.3. Flame-Retardant Mechanism

The flame-retardant mechanism of RPUF composites can be clearly interpreted by analyzing the char residues. The digital photos of char residues of RPUF and its composites are shown in Figure S1 (Supplementary Materials). As can be seen from Figure S1a (Supplementary Materials), the char residues of pure RPUF present many cracks and holes with a discontinue morphology, which is due to the complete combustion of RPUF and release of a large number of gaseous products. After the addition of CEPPG, the char residues of RPUF-2 show a continuous characteristic (See Figure S1b, Supplementary Materials). The char residues become more compact and continuous with the incorporation of EG and P/N compounds (See Figure S1c–f, Supplementary Materials). This phenomenon is attributed to the intumescent effect of EG and the catalytic charring of P/N compounds. In addition, RPUF-4 and RPUF-6 present intact char residues without any cracks in comparison with RPUF-3 and RPUF-5. This result is due to the high content of P of RPUF-4 and RPUF-6. However, the results are opposed to the results from cone calorimeter testing.

In order to study the mechanism for improving the fire safety, the morphologies of the external char residues of RPUF and its composites were investigated by SEM, as presented in Figure S2 (Supplementary Materials). It is observed that plenty of micro-size holes are presented in the external char residues of RPUF (See Figure S2a, Supplementary Materials). However, the char residues of RPUF become continuous with the addition of CEPPG (See Figure S2b, Supplementary Materials), because compact and solid char residues are visible with the incorporation of EG and P/N compounds (See Figure S2c–f, Supplementary Materials). It is noteworthy that RPUF-4 shows smooth and thick external char residues, which is consistent with the digital photo of its char residues.

**Figure 5:**
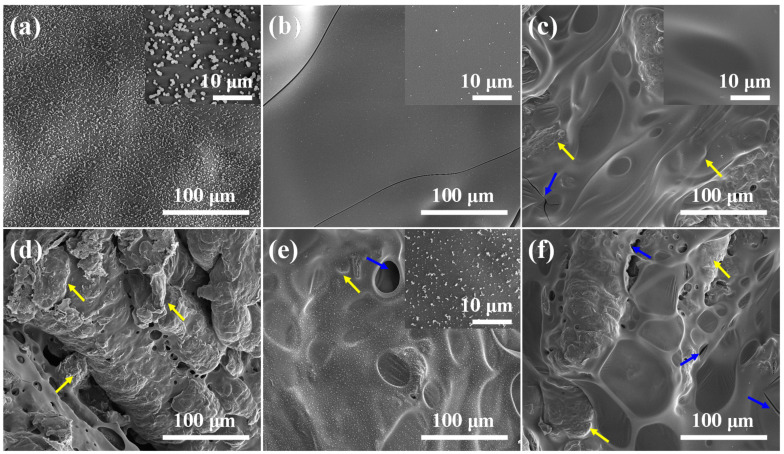
SEM images of internal char residues of (**a**) RPUF-1, (**b**) RPUF-2, (**c**) RPUF-3, (**d**) RPUF-4, (**e**) RPUF-5 and (**f**) RPUF-6 (the arrows refer to the hump and cracks).

To further investigate the mechanism for fire safety enhancement, the morphologies of internal char residues of RPUF and its composites were also performed by SEM, as shown in Figure 5. Apparently, the char residues of pure RPUF contains numerous “popcorn-like” structures (See Figure 5a). Compared to pristine RPUF, RPUF-2 shows a smooth char surface with few micro-size particles. However, some spots also present infinitesimal crack. Upon the incorporation of CEPPG, EG and MP, the RPUF composites, i.e., RPUF-3 and RPUF-5, display smooth and continuous char residues (See Figure 5c,e). This is due to the catalytic charring effect of additives in the condense phase. The char residues of RPUF-3 and RPUF-5 effectively inhibit the production of volatile small molecules and transfer of heat. In addition, numerous “worm-like” layers and cracks are observed from the char residues of RPUF-4 and RPUF-6, especially for RPUF-6. It is contributed to the interpretation that the excessive expansion of EG leading the expanded layered EG tracks cannot be covered by intumescent materials. Therefore, the TSR value of RPUF-4 and RPUF-6 is significantly increased in comparison with that of pure RPUF. Moreover, the overall quality of char residues of RPUF-3 and RPUF-5 is better than that of RPUF-4 and RPUF-6. The phenomenon further indicates the superior synergistic action between EG and MP over that between EG and MPi in promoting charring. The above results demonstrate the significant contribution of internal char residues to improving the flame-retardant property of RPUF materials.

**Figure 6:**
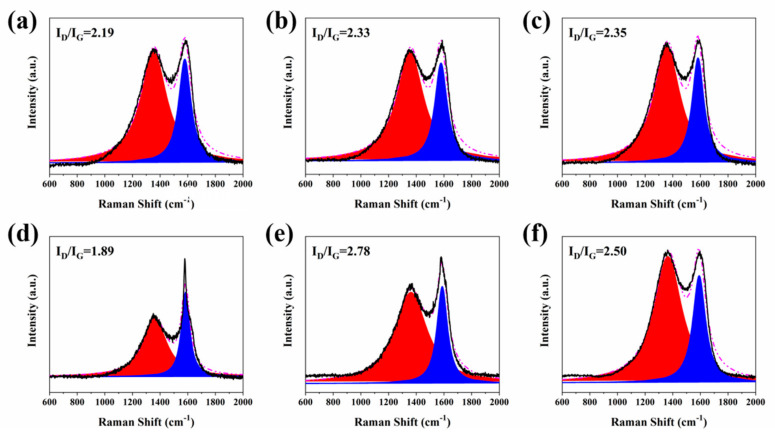
Raman spectra of external char residues of (**a**) RPUF-1, (**b**) RPUF-2, (**c**) RPUF-3, (**d**) RPUF-4, (**e**) RPUF-5 and (**f**) RPUF-6 after cone calorimeter testing.

To further evaluate the quality and graphitization degree of the char residues, the Raman spectra of external char residues of RPUF and its composites are portrayed in Figure 6. It is clear to observe two strong bands, where the first band appears at 1385 cm^−1^ and the second band is located at 1585 cm^−1^, which are named as D and G bands, respectively. The D and G bands are corresponding respectively to the disordered graphite and organized graphitic structures. Generally, the value of area ratio of D band to G band (I_D_/I_G_) is used to estimate the graphitization degree of char residues. It is well known that the lower the value of I_D_/I_G_, the higher the graphitization degree of char residues [52]. Obviously, upon the incorporation of flame-retardant additives, the value of I_D_/I_G_ for the RPUF samples slightly increases, except for RPUF-4. However, the flame retardancy property of all the RPUF samples is better than that of pure RPUF, which indicates that the external char residues have little effect on enhancing the fire safety of RPUF.

**Figure 7:**
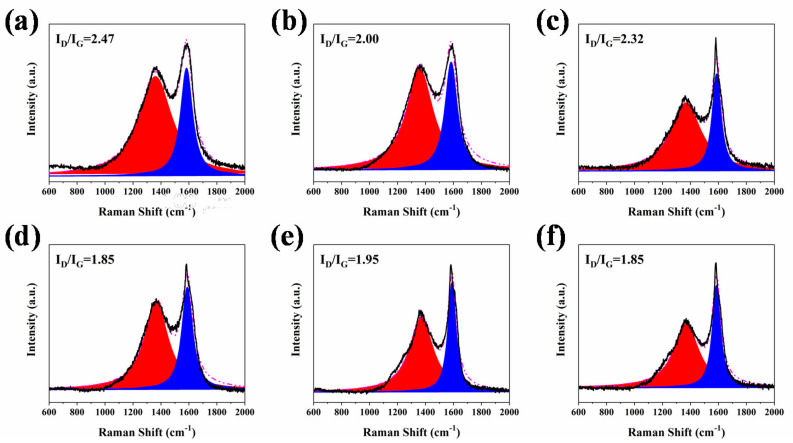
Raman spectra of internal char residues of (**a**) RPUF-1, (**b**) RPUF-2, (**c**) RPUF-3, (**d**) RPUF-4, (**e**) RPUF-5 and (**f**) RPUF-6 after cone calorimeter testing.

To better study the influence of char residue on the flame retardancy property of the RPUF composites, the Raman spectra of internal char residues of all the RPUF samples are presented in Figure 7. It is apparent that after the incorporation of CEPPG, the value of I_D_/I_G_ of RPUF-2 (2.00) is lower than that of RPUF-1 (2.47). The I_D_/I_G_ value further decreases with the addition of EG and P/N compounds. Besides, the I_D_/I_G_ of RPUF-5 is much lower compared to that of RPUF-3, which is caused by the content of P of RPUF-5 being higher than that of RPUF-3. Moreover, the I_D_/I_G_ of RPUF-4 and RPUF-6 are lower than that of RPUF-3 and RPUF-5. This phenomenon is attributed to the lower P valence of MPi, which is beneficial to formation of more char residues. The above results indicate that the internal char residues have significant contribution in the enhanced fire safety of RPUF materials.

**Figure 8:**
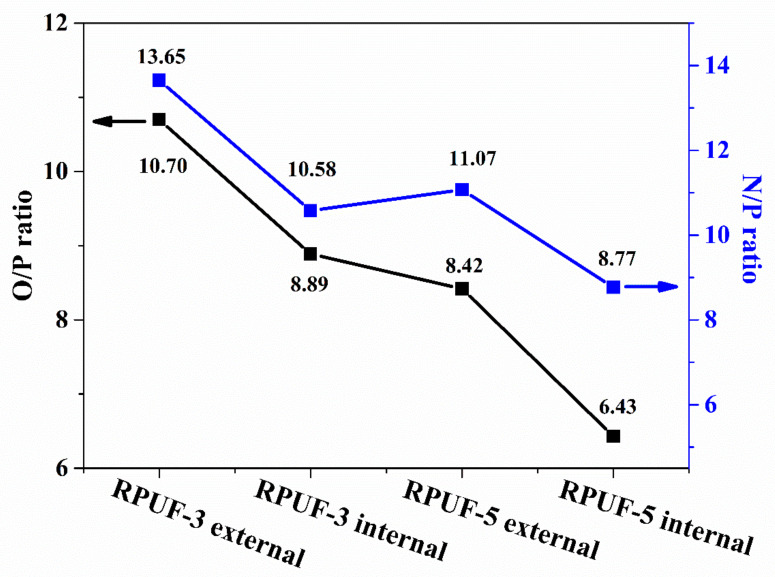
N/P ratio and O/P ratio curves of the char residues of RPUF-3 and RPUF-5.

The XPS survey spectrums of the external and the internal residual chars of RPUF-3 and RPUF-5 are depicted in Figure 8 and Table 4. The concentration of carbon (C) element in the internal char of RPUF-3 and RPUF-5 is higher than that of the external char. However, the concentration of oxygen (O) and nitrogen (N) in the internal char is lower than those in the external char. Different from RPUF-3, RPUF-5 shows a lower P content in the internal char than that in the external char. Besides, both the N/P ratio and the O/P ratio in the external char residue are higher than that in the internal char residue for RPUF-3 and RPUF-5. Besides, the internal char residue of RPUF-5 has the lowest N/P ratio and O/P ratio among all the char residues of RPUF composites, indicating that the internal char residues containing higher content of P are helpful for significant improvement in the char residues of RPUF.

**Figure 9:**
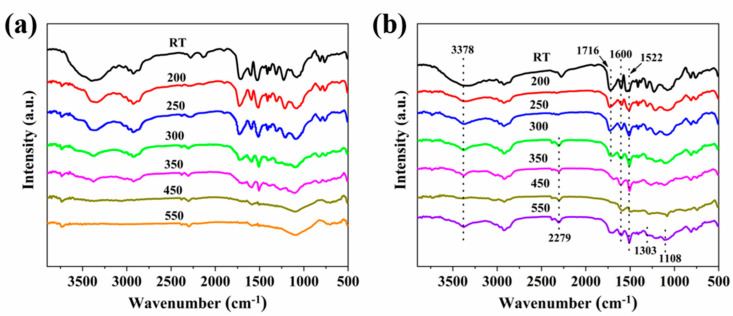
RTIR spectra of (**a**) RPUF-1 and (**b**) RPUF-3.

It is well known that RTIR is an effective technique to study the thermal decomposition of polymeric materials. Therefore, to investigate the thermal-oxidative process of RPUF, RTIR spectroscopy is utilized to investigate the chemical structure change during the temperature-rise process. The thermal degradation behavior at different temperatures of RPUF-1 and RPUF-3 is studied via RTIR measurement, as presented in Figure 9. As can be seen from Figure 9a, some absorption bands emerged at 1522 and 3378 cm^−1^ at room temperature, which are attributed to the bending vibration of N-H and the stretching vibration of N-H, respectively [53]. The band around 1716 cm^−1^ is caused by the stretching vibration of ester C=O, while the asymmetric vibration of the C-O bonds appears at 1224 cm^−1^, which is in accordance with the previous work [54]. The C=O and C-O bonds remain thermally stable until the temperature increases to 350 °C. The peak located at 2279 cm^−1^ is assigned to the unreacted N-CO groups of the isocyanate. Besides, the vibration of C=C bond in the benzene ring appears at 1600 cm^−1^. However, the stretching vibrations corresponding to the N-CO bond disappear at 200 °C, owing to the complete reaction of isocyanate. Moreover, the intensity of all the peaks is gradually decreased with increasing the temperature. This phenomenon is caused by the decomposition of the chemical chain of RPUF materials, especially the degradation of the carbamate bond and the ether bond. There are no apparent absorption bands occurring in the RTIR spectra of RPUF-1 at 500 °C, indicating the total decomposition of RPUF.

As for RPUF-3 (Figure 9b), most of absorption bands are similar to those of the RPUF-1 below 550 °C. However, after the temperature increases to 550 °C, two new absorption peaks are visible in the RTIR spectra. The new peaks are located at 1303 cm^−1^ and 1108 cm^−1^, which correspond to the presence of P=O and O=P−O bonds, respectively. Besides, it is evident that the peaks corresponding to the N-H bond and the phosphorus species are still presented at 550 °C, indicating the incomplete combustion of RPUF-3 due to the protective effect of char residues. The above results indicate that the incorporation of EG and P/N compounds has prevented the thermal degradation of RPUF and improved the fire safety of RPUF.

**Figure 10:**
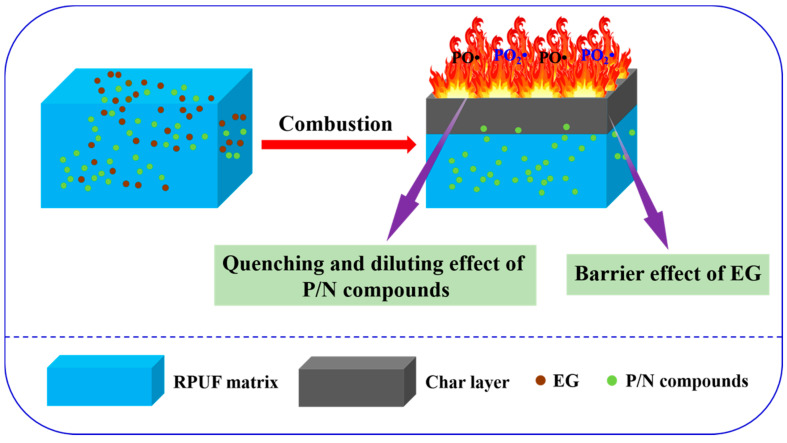
Schematic illustration for flame-retardant mechanism of RPUF composites.

It is well known that the combustibility of polymeric materials is related to the thermal decomposition process, which occurs in the hyperthermal condition. Therefore, the decomposition mechanism of polymeric materials has played a significant role in comprehending the combustion process and the flame retardance of polymeric material. Based on the aforementioned results, the possible flame-retardant mechanism is illustrated in Figure 10. After being ignited, the RPUF thermally degrades into flammable gases, such as CO, HCN, NO and so on. Meanwhile, P/N compounds begin to degrade at low temperature accompanied by generation of free radicals, such as NO_x_•, PO_x_• and so forth. The concentration of combustible gases can be diluted by those free radicals, and PO_x_• can quench the flammable free radicals in the matrix, thus retarding the heat release and smoke generation. As the temperature rises, compact char residues are catalyzed by the synergistic effect between EG and P/N compounds. Meanwhile, “worm-like” char residues are formed in the appearance of RPUF which can act as an excellent physical barrier to isolate the transfer of heat and oxygen during the thermal decomposition process of RPUF. This confirms that char residues further improve the flame retardancy of RPUF. Generally, the diluting and quench effects of P/N compounds in the gas phase together with the excellent catalytic charring effect of EG and P/N compounds in the condense phase have significantly improved the flame retardancy of RPUF.

## 3. Experimental

### 3.1. Raw Materials

Polyether polyol (LY-4110, OH content 430 mg KOH g^−1^, viscosity at 25 °C: 2500 mPa·S), dibutyltin dilaurate (LC), triethylenediamine (A33, 33%) and polyether siloxane surfactant (Si-Oil) were purchased from Jiangsu Luyuan New Materials Co., Ltd. (Nantong, China). Triethanolamine (TEOA), Ethylene glycol, polyaryl polymethylene isocyanate (PM-200, NCO content 30.5–32.0%, viscosity at 25 °C: 150–250 mPa·S), melamine, phosphoric acid (≥85%) phosphate and phosphine were all received from Sinopharm Chemical Reagent Co., Ltd. (Shanghai, China). Expandable graphite (EG) was purchased from Qingdao Tianhe Graphite Company (Particle size: 80 mesh, Qingdao, China). 2-carboxyethyl (phenyl) phosphinic acid (CEPPA) was supported by Jinan Kerry Flame Retardant Technology Co., Ltd. (Jinan, China). Ultrapure water (18.2 MΩ cm^−1^) was obtained from a Milli-Q ultrapure system (Zhengzhou, China). All reagents were used with further purification.

### 3.2. Preparation of 2-Carboxyethyl Phenylphosphinic Acid Glycol Ester (CEPPG)

**Figure 11:**
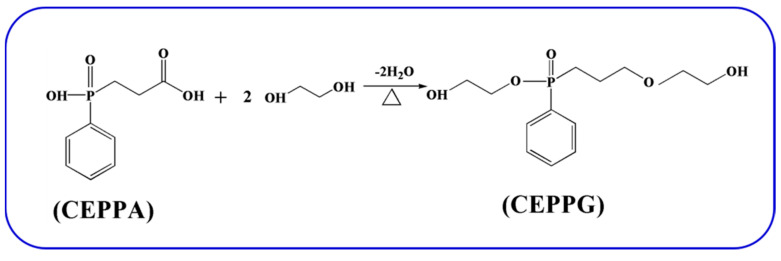
The synthesis route of CEPPG.

CEPPG was synthesized by the esterfication reaction of CEPPA and ethylene glycol according to the previous literature [55]. 0.2 mol CEPPA and 0.8 mol ethylene glycol (the molar ratio of CEPPA and ethylene glycol was 1:4) were cast into a 250 mL three-necked round-bottom flask with a vigorous stirring. The mixture was reacted at 180 °C for 6 h in a nitrogen atmosphere. Subsequently, the mixture was depressed to remove the excess ethylene glycol. Then, a transparent yellow liquid was obtained and denoted as CEPPG. The synthesis route of CEPPG is shown in Figure 11.

### 3.3. Preparation of Melamine Phosphate (MP) and Melamine Phosphite (MPi)

**Figure 12:**
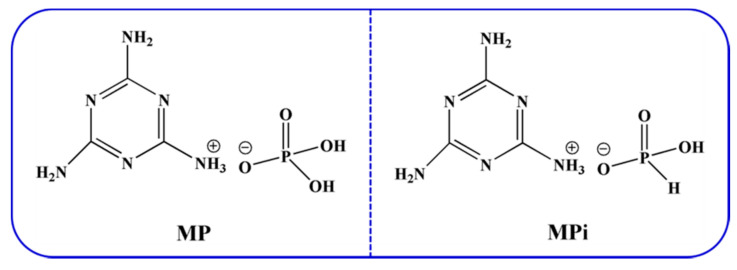
The chemical structures of Melamine Phosphate (MP) and Melamine Phosphite (MPi).

37.84 g melamine and 750 mL ultrapure water were added into a 1000 mL three-necked round-bottomed flask with mechanical stirring, and then heated to 95 °C. After continuous mechanical stirring for 30 min until the melamine was completely dissolved, 34.6 g phosphate was added dropwise into the above solution, and the mixture was kept at 95 °C for 1 h with continuous mechanical stirring. The precipitate was filtered and washed with ultrapure water, and then dried at 70 °C for 24 h. The as-prepared solids were labelled as melamine phosphate (MP). The preparation process for the melamine phosphite (MPi) was similar to that of MP, excepted that phosphite was used to replace phosphate [29]. The chemical structures of MP and MPi are presented in Figure 12.

### 3.4. Fabrication of Flame Retardant RPPU Composites

RPUF and its flame retardant composites were prepared by the one-pot and free-foaming method. The components LY4110, CEPPG, A33, water, silicone surfactant and TEOA were firstly mixed in a 500 mL plastic breaker with a mechanical stirring for 15 s. PM-200, EG, MP and MPi were mixed in another 250 mL plastic breaker with a mechanical for 15 s to achieve a uniform slurry. Subsequently, the slurry was added into the 500 mL plastic breaker with vigorous stirring for 10 s. Then, the above mixture was immediately poured into a plastic box (190×130×90 mm^3^). Finally, the obtained samples were kept in an oven at 70 °C for 12 h to guarantee the complete reaction. The formulations of RPUF samples were showed in Table 5.

### 3.5. Instruments and Measurements

Thermogravimetric analysis (TGA) was performed using a Q5000 analyzer (TA Co., New Castle, DE, USA) [38]. The RPUF samples were heated from room temperature to 800 °C at a linear heating of 20 °C min^−1^ in ambient air. The UL-94 vertical burning test was conducted using a CZF-II horizontal and vertical burning tester (Jiang Ning Analysis Instrument Co, Ltd, Nanjing, China). The dimension used in the vertical burning test was 127 × 12.7 × 3 mm^3^. The combustion properties of RPUF and its composites were investigated via a cone calorimeter (TESTech, Suzhou, China) according to ISO 5660. The specimens with size of 100 × 100 × 3 mm^3^ were radiated by a heat flux of 35 kW m^−2^. The specimen was placed in an aluminum foil. All these procedures were performed three times for each specimen. The morphology of char residues of RPUF and its composites were characterized by a scanning electron microscope (SEM) (AMRAY1000B, Beijing R&D Center of the Chinese Academy of Sciences, Beijing, China). Before SEM evaluation, the samples were sputter-coated with gold. Raman spectra of RPUF and its composites were performed with using a SPEX-1403 laser Raman spectrometer (SPEX Co., Metuchen, NJ, USA) with an excitation wavelength of 514 nm. X-ray photoelectron spectroscopy (XPS) of RPUF and its composites were performed by a VG Escalab Mark II spectrometer (Thermo Fisher Scientific Ltd., Waltham, MA, USA), using Al K, an excitation radiation (ht = 1486.6 eV). The real-time Fourier transform infrared (RTIR) spectrums of RPUF and its composites were investigated with a linear heating rate of 20 °C min^−1^ from room temperature to 600 °C.

## 4. Conclusions

In this work, EG and P/N compounds were successfully incorporated into RPUF via a simple free foaming approach. The fire safety of RPUF composites was investigated by various techniques. The results indicated that the thermal stability of RPUF was significantly improved with the addition of EG and P/N compounds, and all the RPUF composites containing EG and MP passed the UL-94 V-0 rating. With the addition of 22.4 wt.% P/N compounds and 3.2 wt.% EG, the THR and TSR were decreased by 29.6% and 32.4%, respectively. The quenching and diluting effect of P/N compounds combined with the excellent catalytic charring of EG led to reduced heat release and smoke generation. This work provides a novel method for improving the fire safety of RPUF.

## Figures and Tables

**Table 1:** The related thermal data of RPUF and its composites under air condition.

Sample No.	T_−5_ (°C)	T_−50_ (°C)	T_max_ (°C)		Residues at 750 °C (wt.%)
			Step 1	Step 2	
RPUF-1	260.8	383.4	297.4	604.6	1.41
RPUF-2	220.4	504.6	310.2	590.3	8.74
RPUF-3	240.6	505.4	294.1	556.5	5.32
RPUF-4	245.1	437.9	294.4	564.4	4.18
RPUF-5	236.6	497.7	294.3	550.6	5.65
RPUF-6	236.2	486.4	297.2	565.4	4.33

Notes: T_−5_ means the temperature at 5% weight loss; T_−50_ denotes the temperature at 50% weight loss; T_max_ indicates the temperature corresponding to the maximum weight loss rate.

**Table 2:** The vertical burning data of RPUF and its composites.

Sample No.	t_1_/t_2_ (s)	Ignite the Cotton	Rating
RPUF-1	―	No	NR
RPUF-2	19.7/0.0	No	V-1
RPUF-3	6.9/0.0	No	V-0
RPUF-4	15.7/0.0	No	V-1
RPUF-5	2.4/0.0	No	V-0
RPUF-6	17.8/0.0	No	V-1

Note: t_1_ means first burning time; t_2_ represents second burning time; NR denotes no rating.

**Table 3:** The data of RPUF and its composites after cone calorimeter test.

Sample No.	TTI (s)	PHRR (kW m^−2^)	THR (MJ m^−2^)	PSRR (m^2^ s^−1^)	TSR (m^2^ m^−2^)
Error	±1	±21	±2	±0.02	±696
RPUF-1	5	312	27.0	0.24	3698
RPUF-2	6	287	18.8	0.42	3255
RPUF-3	4	233	19.0	0.38	2489
RPUF-4	4	270	23.6	0.31	3957
RPUF-5	3	235	20.6	0.35	3525
RPUF-6	3	262	27.0	0.34	4248

Notes: TTI—Time to ignition; PHRR—Peak of heat release rate; THR—Total heat release; PSPR—Peak of smoke production rate (SPR).

**Table 4:** The XPS data of RPUF-3 and RPUF-5.

Sample No.		C (At.%)	O (At.%)	N (At.%)	P (At.%)	N/P	O/P
RPUF-3	External char	79.22	8.77	11.19	0.82	13.65	10.70
	Internal char	82.59	7.56	8.99	0.85	10.58	8.89
RPUF-5	External char	75.43	10.1	13.28	1.2	11.07	8.42
	Internal char	84.45	6.17	8.42	0.96	8.77	6.43

**Table 5:** Formulations of RPUF and its composites.

Sample No.	RPUF-1	RPUF-2	RPUF-3	RPUF-4	RPUF-5	RPUF-6
LY4110 (g)	100	54.94	54.94	54.94	54.94	54.94
CEPPG (g)	0	45.06	45.06	45.06	45.06	45.06
A33 (g)	1	1	1	1	1	1
LC (g)	0.5	0.5	0.5	0.5	0.5	0.5
Water (g)	2	2	2	2	2	2
Si-Oil (g)	2	2	2	2	2	2
TEOA (g)	3	3	3	3	3	3
PM-200 (g)	150	126.3	126.3	126.3	126.3	126.3
EG (g)	0	0	7.5	7.5	8.82	8.82
MP (g)	0	0	7.5	0	8.82	0
Mpi (g)	0	0	0	7.5	0	8.82
P/N compounds wt.%	0	19.2	22.4	22.4	23.0	23.0
EG wt.%	0	0	3.2	3.2	3.8	3.8

## Supplementary Materials

The following are available online. Figure S1: Digital photos of char residues of (a) RPUF-1, (b) RPUF-2, (c) RPUF-3, (d) RPUF-4, (e) RPUF-5 and (f) RPUF-6 after cone calorimeter test, Figure S2: SEM images of external char residues of (**a**) RPUF-1, (**b**) RPUF-2, (**c**) RPUF-3, (**d**) RPUF-4, (**e**) RPUF-5 and (**f**) RPUF-6.

## References

[1] Borreguero A.M., Velencoso M.M., Rodriguez J.F., Serrano A., Carrero M.J., Ramos M.J. (2019). Synthesis of aminophosphonate polyols and polyurethane foams with improved fire retardant properties. J. Appl. Polym. Sci..

[2] Luo F., Wu K., Li D., Zheng J., Guo H., Zhao Q., Lu M. (2017). A novel intumescent flame retardant with nanocellulose as charring agent and its flame retardancy in polyurethane foam. Polym. Compos..

[3] Chiu S.H., Wu C.L., Lee H.T., Gu J.H., Suen M.C. (2016). Synthesis and characterisation of novel flame retardant polyurethanes containing designed phosphorus units. J. Polym. Res..

[4] Liu C., Fang Y.F., Miao X.M., Pei Y.B., Yan Y., Xiao W.J., Wu L.B. (2019). Facile fabrication of superhydrophobic polyurethane sponge towards oil water separation with exceptional flame-retardant performance. Sep. Purif. Technol..

[5] Yang H.Y., Liu H.Y., Jiang Y.P., Chen M.F., Wan C.J. (2019). Density effect on flame retardancy, thermal degradation, and combustibility of rigid polyurethane foam modified by expandable graphite or ammonium polyphosphate. Polymers.

[6] Gao M., Li J.F., Zhou X. (2019). A flame retardant rigid polyurethane foam system including functionalized graphene oxide. Polym. Compos..

[7] Xu D.F., Yu K.J., Qian K. (2017). Effect of tris(1-chloro-2-propyl)phosphate and modified aramid fiber on cellular structure, thermal stability and flammability of rigid polyurethane foams. Polym. Degrad. Stabil..

[8] Yuan Y., Yang H.Y., Yu B., Shi Y.Q., Wang W., Song L., Hu Y., Zhang Y.M. (2016). Phosphorus and nitrogen-containing polyols: Synergistic effect on the thermal property and flame retardancy of rigid polyurethane foam composites. Ind. Eng. Chem. Res..

[9] Zhang M., Luo Z.Y., Zhang J.W., Chen S.G., Zhou Y.H. (2015). Effects of a novel phosphorus-nitrogen flame retardant on rosin-based rigid polyurethane foams. Polym. Degrad. Stabil..

[10] Zhang L., Zhang M., Zhou Y., Hu L. (2013). The study of mechanical behavior and flame retardancy of castor oil phosphate-based rigid polyurethane foam composites containing expanded graphite and triethyl phosphate. Polym. Degrad. Stabil..

[11] Xu W., Wang G., Zheng X. (2015). Research on highly flame-retardant rigid PU foams by combination of nanostructured additives and phosphorus flame retardants. Polym. Degrad. Stabil..

[12] Chen Y., Li L., Qi X., Qian L. (2019). The pyrolysis behaviors of phosphorus-containing organosilicon compound modified APP with different polyether segments and their flame retardant mechanism in polyurethane foam. Compos. Part. B Eng..

[13] Chen Y., Li L., Qian L. (2018). The pyrolysis behaviors of phosphorus-containing organosilicon compound modified ammonium polyphosphate with different phosphorus-containing groups, and their different flame-retardant mechanisms in polyurethane foam. RSC Adv..

[14] Zheng X., Wang G., Xu W. (2014). Roles of organically-modified montmorillonite and phosphorous flame retardant during the combustion of rigid polyurethane foam. Polym. Degrad. Stabil..

[15] Xu Z.B., Kong W.W., Zhou M.X., Peng M. (2010). Eeffect of surface modification of montmorillonite on the properties of rigid polyurethane foam composites. Chin. J. Polym. Sci..

[16] Xu W.Z., Wang G.S., Xu J.Y., Liu Y.C., Chen R., Yan H.Y. (2019). Modification of diatomite with melamine coated zeolitic imidazolate framework-8 as an effective flame retardant to enhance flame retardancy and smoke suppression of rigid polyurethane foam. J. Hazard. Mater..

[17] Wang J., Ma C., Mu X., Cai W., Liu L., Zhou X., Hu W., Hu Y. (2018). Construction of multifunctional MoSe_2_ hybrid towards the simultaneous improvements in fire safety and mechanical property of polymer. J. Hazard. Mater..

[18] Xu Q.W., Zhai H.M., Wang G.J. (2015). Mechanism of smoke suppression by melamine in rigid polyurethane foam. Fire Mater..

[19] Akdogan E., Erdem M., Ureyen M.E., Kaya M. (2020). Rigid polyurethane foams with halogen-free flame retardants: Thermal insulation, mechanical, and flame retardant properties. J. Appl. Polym. Sci..

[20] Chai H., Duan Q.L., Jiang L., Sun J.H. (2019). Effect of inorganic additive flame retardant on fire hazard of polyurethane exterior insulation material. J. Therm. Anal. Calorim..

[21] Wang Y.T., Wang F., Dong Q.X., Xie M.C., Liu P., Ding Y.F., Zhang S.M., Yang M.S., Zheng G.Q. (2017). Core-shell expandable graphite @ aluminum hydroxide as a flame-retardant for rigid polyurethane foams. Polym. Degrad. Stabil..

[22] Wu S., Deng D., Zhou L., Zhang P., Tang G. (2019). Flame retardancy and thermal degradation of rigid polyurethane foams composites based on aluminum hypophosphite. Mater. Res. Express.

[23] Xu W.Z., Liu L., Wang S.Q., Hu Y. (2015). Synergistic effect of expandable graphite and aluminum hypophosphite on flame-retardant properties of rigid polyurethane foam. J. Appl. Polym. Sci..

[24] Tang Q., Song Y., He J., Yang R. (2014). Synthesis and characterization of inherently flame-retardant and anti-dripping thermoplastic poly(imides-urethane)s. J. Appl. Polym. Sci..

[25] Jia D., Guo X., He J., Yang R. (2019). An anti-melt dripping, high char yield and flame-retardant polyether rigid polyurethane foam. Polym. Degrad. Stabil..

[26] Huang Y., Jiang S., Liang R., Liao Z., You G. (2019). A green highly-effective surface flame-retardant strategy for rigid polyurethane foam: Transforming UV-cured coating into intumescent self-extinguishing layer. Compos. Part. A Appl. Sci. Manuf..

[27] Xue B., Qin R., Shao M., Li S., Niu M. (2019). Improving the flame retardancy of PET fiber by constructing the carbon microspheres based melamine polyphosphate powder. J. Text. Inst..

[28] Thirumal M., Khastgir D., Nando G.B., Naik Y.P., Singha N.K. (2010). Halogen-free flame retardant PUF: Effect of melamine compounds on mechanical, thermal and flame retardant properties. Polym. Degrad. Stabil..

[29] Yang H., Song L., Tai Q., Wang X., Yu B., Yuan Y., Hu Y., Yuen R.K.K. (2014). Comparative study on the flame retarded efficiency of melamine phosphate, melamine phosphite and melamine hypophosphite on poly(butylene succinate) composites. Polym. Degrad. Stabil..

[30] Cheng J.J., Qu W.J., Sun S.H. (2019). Mechanical properties improvement and fire hazard reduction of expandable graphite microencapsulated in rigid polyurethane foams. Polym. Compos..

[31] Wang G., Bai S. (2017). Synergistic effect of expandable graphite and melamine phosphate on flame-retardant polystyrene. Appl. Polym. Sci..

[32] Pang X.Y., Xin Y.P., Shi X.Z., Xu J.Z. (2019). Effect of different size-modified expandable graphite and ammonium polyphosphate on the flame retardancy, thermal stability, physical, and mechanical properties of rigid polyurethane foam. Polym. Eng. Sci..

[33] Chen Y., Luo Y., Guo X., Chen L., Xu T., Jia D. (2019). Structure and flame-retardant actions of rigid polyurethane foams with expandable graphite. Polymers.

[34] Xi W., Qian L., Chen Y., Wang J., Liu X. (2015). Addition flame-retardant behaviors of expandable graphite and bis (2-hydroxyethyl) aminol-methyl-phosphonic acid dimethyl ester in rigid polyurethane foams. Polym. Degrad. Stabil..

[35] Wang S., Qian L., Xin F. (2018). The synergistic flame-retardant behaviors of pentaerythritol phosphate and expandable graphite in rigid polyurethane foams. Polym. Compos..

[36] Liu X., Sun J., Zhang S., Guo J., Tang W., Li H., Gu X. (2019). Effects of carboxymethyl chitosan microencapsulated melamine polyphosphate on the flame retardancy and water resistance of thermoplastic polyurethane. Polym. Degrad. Stabil..

[37] Feng F., Qian L. (2014). The flame retardant behaviors and synergistic effect of expandable graphite and dimethyl methylphosphonate in rigid polyurethane foams. Polym. Compos..

[38] Shi Y., Liu C., Liu L., Fu L., Yu B., Lv Y., Yang F., Song P. (2019). Strengthening, toughing and thermally stable ultra-thin MXene nanosheets/polypropylene nanocomposites via nanoconfinement. Chem. Eng. J..

[39] Shi Y.Q., Gui Z., Yuan B.H., Hu Y., Zheng Y.Y. (2018). Flammability of polystyrene/aluminim phosphinate composites containing modified ammonium polyphosphate. J. Therm. Anal. Calorim..

[40] Han S., Zhu X., Chen F., Chen S., Liu H. (2020). Flame-retardant system for rigid polyurethane foams based on diethyl bis (2-hydroxyethyl) aminomethylphosphonate and in-situ exfoliated clay. Polym. Degrad. Stabil..

[41] Singh H., Jain A.K. (2009). Ignition, combustion, toxicity, and fire retardancy of polyurethane foams: A comprehensive review. J. Appl. Polym. Sci..

[42] Levchik S.V., Weil E.D. (2004). Thermal decomposition, combustion and fire-retardancy of polyurethanes—A review of the recent literature. Polym. Int..

[43] Du J.Z., Alain G., Zeng H.Y., Feng B., Xu S., Zhou E.G., Shi X.K., Jin L. (2020). Electron beam irradiation crosslinking and flame retardant of ethylene vinyl acetate using melamine-formaldehyde microencapsulated layered double hydroxides. J. Nanosci. Nanotechnol..

[44] Xiao F., Wu K., Luo F.B., Guo Y.Y., Zhang S.H., Du X.X., Zhu Q.Q., Lu M.G. (2017). An efficient phosphonate-based ionic liquid on flame retardancy and mechanical property of epoxy resin. J. Mater. Sci..

[45] Latha G., Natarajan M., Murugavel S.C. (2016). Synthesis and characterization of cardo-based phosphorous-containing flame-retardant aromatic polyesters. High. Perform. Polym..

[46] Shi Y., Wang L., Fu L., Liu C., Yu B., Yang F., Hu Y. (2019). Sodium alginate-templated synthesis of g-C_3_N_4_/carbon spheres/Cu ternary nanohybrids for fire safety application. J. Colloid Interface Sci..

[47] Shi Y., Liu C., Duan Z., Yu B., Liu M., Song P. (2020). Interface engineering of MXene towards super-tough and strong polymer nanocomposites with high ductility and excellent fire safety. Chem. Eng. J..

[48] Shi Y., Yu B., Duan L., Gui Z., Wang B., Hu Y., Yuen R.K.K. (2017). Graphitic carbon nitride/phosphorus-rich aluminum phosphinates hybrids as smoke suppressants and flame retardants for polystyrene. J. Hazard. Mater..

[49] Akech S.R.O., Harrison O., Saha A. (2018). Removal of a potentially hazardous chemical, tetrakis (hydroxymethyl) phosphonium chloride from water using biochar as a medium of adsorption. Environ. Technol. Innov..

[50] Yuan Y., Yu B., Shi Y.Q., Ma C., Song L., Hu W.Z., Hu Y. (2018). Highly efficient catalysts for reducing toxic gases generation change with temperature of rigid polyurethane foam nanocomposites: A comparative investigation. Compos. Part. A Appl. Sci. Manuf..

[51] Wang L., Tawiah B., Shi Y., Cai S., Rao X., Liu C., Yang Y., Yang F., Yu B., Liang Y. (2019). Highly effective flame-retardant rigid polyurethane foams: Fabrication and applications in inhibition of coal combustion. Polymers.

[52] Yuan Y., Ma C., Shi Y.Q., Song L., Hu Y., Hu W.Z. (2018). Highly-efficient reinforcement and flame retardancy of rigid polyurethane foam with phosphorus-containing additive and nitrogen-containing compound. Mater. Chem. Phys..

[53] Hejna A., Kirpluks M., Kosmela P., Cabulis U., Haponiuk J., Piszczyk L. (2017). The influence of crude glycerol and castor oil-based polyol on the structure and performance of rigid polyurethane-polyisocyanurate foams. Ind. Crop. Prod..

[54] Jiao L., Xiao H., Wang Q., Sun J. (2013). Thermal degradation characteristics of rigid polyurethane foam and the volatile products analysis with TG-FTIR-MS. Polym. Degrad. Stabil..

[55] Li L.J., Duan R.T., Zhang J.B., Wang X.L., Chen L., Wang Y.Z. (2013). Phosphorus-containing poly (ethylene terephthalate): Solid-state polymerization and its sequential distribution. Ind. Eng. Chem. Res..

