# Supplementary material for: Enhanced Fire Safety of Rigid Polyurethane Foam via Synergistic Effect of Phosphorus/Nitrogen Compounds and Expandable Graphite
Source: Molecules. 2020 Oct 15;25(20):4741. doi: 10.3390/molecules25204741 (PMC7587603; doi:10.3390/molecules25204741)
Supplement: Supplementary file 1 [file molecules-25-04741-s001.pdf]

molecules

Supplementary Information

Enhanced Fire Safety of Rigid Polyurethane Foam via Synergistic Effect of Phosphorus/Nitrogen Compounds and Expandable Graphite

Chuan Liu [1,†], Ping Zhang [2,†], Yongqian Shi [1,*], Xiaohui Rao [1], Suncheng Cai [1], Libi Fu [3], Yuezhan Feng [4], Liancong Wang [5,*], Xueqin Zheng [6] and Wei Yang [7,*]

[1] College of Environment and Resources, Fuzhou University, 2 Xueyuan Road, Fuzhou 350116, China; liuchuanll@163.com (C.L.); Rxh123456789m88@163.com (X.R.); dandy_cai@163.com (S.C.)

[2] State key Laboratory of Enviromental Friendly Energy Materials & Department of Materials, Southwest University of Science and Technology, Mianyang 621010, China; pingzhang@swust.edu.cn

[3] College of Civil Engineering, Fuzhou University, 2 Xueyuan Road, Fuzhou 350116, China; fulibi@fzu.edu.cn

[4] Key Laboratory of Materials Processing and Mold Ministry of Education, National Engineering Research Center for Advanced Polymer Processing Technology, Zhengzhou University, Zhengzhou 450002, China; yzfeng@zzu.edu.cn

[5] State Key Laboratory of Coal Mine Safety Technology, CCTEG Shenyang Research Institute, Fushun 113122, China

[6] College of Safety and Environment, Fujian Chuanzheng Communications College, 80 Shoushan Road, Fuzhou 350007, China; zhengxueqin815@foxmail.com

[7] School of Energy, Materials and Chemical Engineering, Hefei University, Hefei 230601, China

* Correspondence: shiyq1986@fzu.edu.cn (Y.S.); wangliancong-0829@163.com (L.W.); yangwei@hfuu.edu.cn (W.Y.)

† These authors contributed equally to this work.

Additional Supporting Data

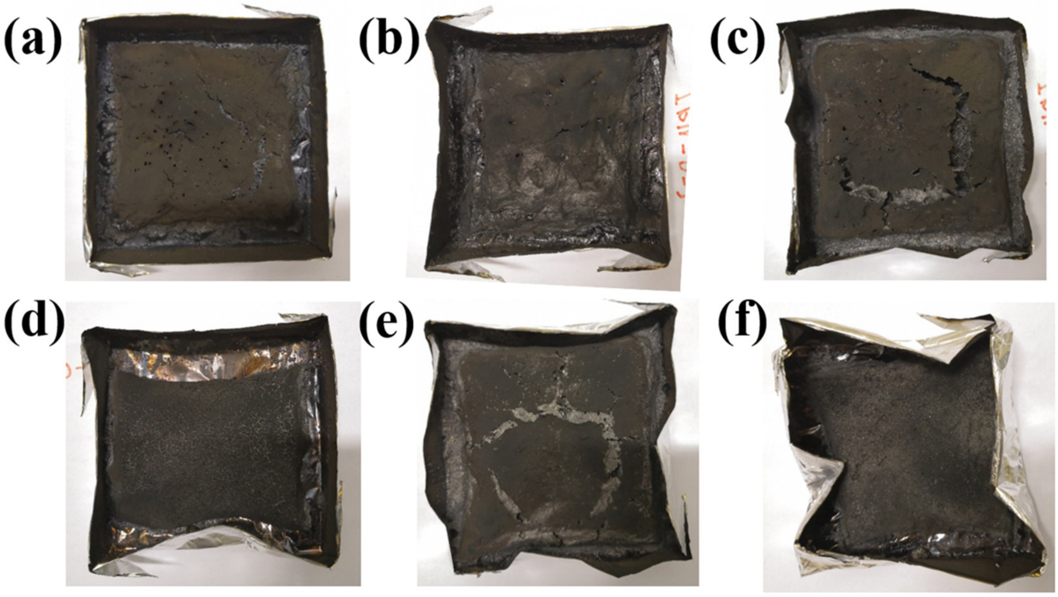

Figure S1. Digital photos of char residues of (**a**) RPUF-1, (**b**) RPUF-2, (**c**) RPUF-3, (**d**) RPUF-4, (**e**) RPUF-5 and (**f**) RPUF-6 after cone calorimeter test.

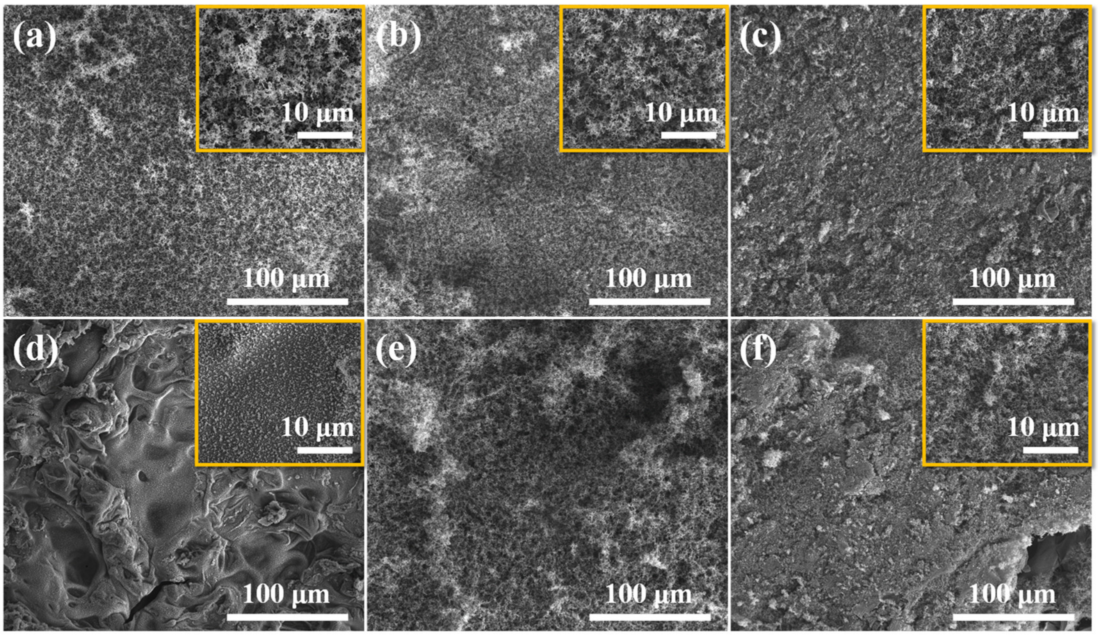

Figure S2. SEM images of external char residues of (**a**) RPUF-1, (**b**) RPUF-2, (**c**) RPUF-3, (**d**) RPUF-4, (**e**) RPUF-5 and (**f**) RPUF-6.

© 2020 by the authors. Licensee MDPI, Basel, Switzerland. This article is an open access article distributed under the terms and conditions of the Creative Commons Attribution (CC BY) license (http://creativecommons.org/licenses/by/4.0/).
